# *In Vitro* Dose-Dependent Inhibition of the Intracellular Spontaneous Calcium Oscillations in Developing Hippocampal Neurons by Ketamine

**DOI:** 10.1371/journal.pone.0059804

**Published:** 2013-03-28

**Authors:** Lining Huang, Yue Liu, Pei Zhang, Rongtian Kang, Ya Liu, Xuze Li, Lijun Bo, Zhenming Dong

**Affiliations:** Department of Anesthesiology, the Second Hospital of Hebei Medical University, Shijiazhuang, Hebei Province, China; University of Maribor, Slovenia

## Abstract

Spatial and temporal abnormalities in the frequency and amplitude of the cytosolic calcium oscillations can impact the normal physiological functions of neuronal cells. Recent studies have shown that ketamine can affect the growth and development and even induce the apoptotic death of neurons. This study used isolated developing hippocampal neurons as its study subjects to observe the effect of ketamine on the intracellular calcium oscillations in developing hippocampal neurons and to further explore its underlying mechanism using Fluo-4-loaded laser scanning confocal microscopy. Using a semi-quantitative method to analyze the spontaneous calcium oscillatory activities, a typical type of calcium oscillation was observed in developing hippocampal neurons. In addition, the administration of NMDA (N-Methyl-D-aspartate) at a concentration of 100 µM increased the calcium oscillation amplitude. The administration of MK801 at a concentration of 40 µM inhibited the amplitude and frequency of the calcium oscillations. Our results demonstrated that an increase in the ketamine concentration, starting from 30 µM, gradually decreased the neuronal calcium oscillation amplitude. The inhibition of the calcium oscillation frequency by 300 µM ketamine was statistically significant, and the neuronal calcium oscillations were completely eliminated with the administration of 3,000 µM Ketamine. The administration of 100, 300, and 1,000 µM NMDA to the 1 mM ketamine-pretreated hippocampal neurons restored the frequency and amplitude of the calcium oscillations in a dose-dependent manner. In fact, a concentration of 1,000 µM NMDA completely reversed the decrease in the calcium oscillation frequency and amplitude that was induced by 1 mM ketamine. This study revealed that ketamine can inhibit the frequency and amplitude of the calcium oscillations in developing hippocampal neurons though the NMDAR (NMDA receptor) in a dose-dependent manner, which might highlight a possible underlying mechanism of ketamine toxicity on the rat hippocampal neurons during development.

## Introduction

In the central nervous system, Ca^2+^, as a ubiquitous cell messenger that participates in the regulation of neurotransmitter release and nerve excitability, is closely associated with cellular differentiation and migration, synaptic plasticity, neurite growth, neurotransmitter release, and neuronal apoptosis [1]–[3]. Under normal physiological conditions, there is a spatiotemporal combination pattern in intracellular calcium signaling transduction: the transduction starts from a local cytosolic Ca^2+^ concentration elevation and is then transmitted in the cells in a recurrent and transient pattern. This periodical appearance of calcium peaks is called calcium oscillation. Neuronal calcium oscillation activities are featured with a diversity of temporal and spatial fluctuation patterns in the frequency and amplitude of cytosolic calcium concentration fluctuations, which can affect the physiological functions of neuronal cells, such as proliferation and development, long-term potentiation, long-term depression, contraction, and secretion [4]–[6]. When the frequency and amplitude of cytosolic calcium oscillations are beyond the normal temporal and spatial ranges, the physiological functions of neuronal cells, such as proliferation and development, will be inevitably affected. Studies have showed that the N-methyl-D-aspartate receptor (NMDAR) plays important roles in the entire neuronal development process, including proliferation, differentiation, and synaptic plasticity [7], and is closely associated with intracellular Ca^2+^ levels in neurons [8], [9]. Ketamine, a commonly used intravenous anesthetic in clinical anesthesia, exerts its function as a non-competitive NMDAR antagonist. Recent studies have showed that ketamine can affect neuronal growth and development and even induce apoptosis [10]–[12]. However, the effects of ketamine and NMDA on calcium oscillations in hippocampal neurons during development still remain unclear. Using isolated developing hippocampal neurons as study subjects, this study attempted to investigate the effects of ketamine and NMDA on calcium oscillations in developing hippocampal neurons and their underlying mechanism. This analysis, which used Fluo-4-loaded laser scanning confocal microscopy, was performed to further elucidate the mechanism of ketamine toxicity on hippocampal neurons during development.

## Materials and Methods

### Ethics Statement

Animal experiments were performed according to the regulations of laboratory animal management promulgated by the Ministry of Science and Technology of the People’s Republic of China [1988] No. 134, which conforms to the internationally recognized NIH guidance for care and use of laboratory animals.

### Main Chemicals and Equipments

DMEM medium, Fetal bull serum(FBS), B27 and N2 serum-free supplements were purchased from Gibco BRL (Gaithersburg, MD, USA). Matrigel basement membrane matrix were purchased from Becton, Dickinson and Company(BD, USA). Accutase cell dissociation reagent and N-methyl-D-aspartate(NMDA) were getten from Sigma-Aldrich Company(Sigma,USA). Anti-rat monoclonal antibody against β-tublinШ was purchased from Millipore Corporation(Millipore, USA). Neurobasal medium, Fluo-4 acetoxymethyl and HEPES buffer solution were from Invitrogen Corporation(Invitrogen, USA). MK801 was purchased from Sigma corporation(Sigma, USA) and ENZO corporation(ENZO, USA), respectively. Pure Ketamine was obtained from Jiangsu Hengrui Medicine Co., Ltd. TCS-SP2 Confocal laser scanning microscope and LeicaLCSLite Image analysis software were made in Leica corporation(Leica,Germany).

### Cell Culture

A modified cell culture method was adopted. The bilateral hippocampal tissues were exposed and removed from 24-h postnatal Sprague-Dawley rats and disinfected with 75% alcohol. After the blood vessels and meninges were removed, the hippocampal tissues were cut into pieces that were as small as possible. These pieces were then centrifuged at 800 rpm for 5 min. After the supernatant was discarded, a volume of Accutase that was three times the volume of the hippocampal tissues was added. The samples were then placed in an incubator for 10–20 min for digestion. During the digestion, the tissues were vortexed several times. DMEM with 10% FBS was then added to terminate the digestion process. After being thoroughly dispersed by pipetting, the tissues were filtered through a 200-mesh copper filter. The filtered cells were collected and centrifuged at 800 rpm for 5 min. Subsequently, the supernatant was discarded, DMEM with 10% FBA was added, and the cells were dispersed to obtain a cell suspension. After being transferred into plastic culture flasks, the cells were cultured for 1 h in an incubator. The neurons were harvested using a differential adhesion method. After dispersion, the cells were stained with 0.4% Trypan blue to count the live cells. Following the adjustment of the cell density of the suspension to 1×10^6^ cells/mL, the cells were inoculated onto Matrigel-coated coverslips in 24-well culture plates with a 150 µL cell suspension per slide. The inoculated 24-well plates were placed in a moisture box containing deionized water and incubated in a CO_2_ incubator. After 24 h, the culture medium was changed to neurobasal medium containing 1% N2 and 2% B27. The half culture medium was changed every 2 days until day 5.

### Calcium Imaging

Fluo-4 AM was diluted using Krebs-Ringer solution (150 mM NaCl, 5 mM KCl, 2 mM CaCl_2_, 1 mM MgCl_2_, 10 mM HEPES, and 10 M D-glucose) into a 4 µM Fluo-4 AM working solution. The hippocampal neurons cultured on the coverslips for 5 days were washed three times with Krebs-Ringer solution for 5 min. The Fluo-4 working solution was added to the primary hippocampal neurons; the cells were then incubated at 37°C for 30 min. The cells were again washed three times with Krebs-Ringer solution for 5 min and then incubated in Krebs-Ringer solution for 15 min to remove any non-specific staining on the cell surface.

The cell culture coverslips incubated with Fluo-4AM were placed in a special perfusion chamber of the laser scanning confocal microscope. After the perfusion apparatus was installed, the amount of injected and aspirated liquid was balanced at a speed of 2–3 mL/min; the temperature of the water bath was maintained at 37°C. The focal plane was adjusted to clearly display the hippocampal neurons under the laser scanning confocal microscope. The fluorescence was excited by an argon ion laser with an excitation wavelength of 488 nm and an emission wavelength of 530 nm. The cells were scanned in an XYT-plane fashion under the laser scanning confocal microscope using the Time Series program. The scanning frequency was 1 time/s, and the changes in the fluorescence were recorded through continuous scanning. After perfusion, the average fluorescence intensity of a cell in the XYT-plane was calculated and used to represent the [Ca^2+^]i. As the control measurement before the drug intervention, the neurobasal medium or the neurobasal medium containing the pretreatment drug was perfused for 2 min. The medium was then changed to the neurobasal medium containing the intervening drug (the pretreatment drug and the intervening drug were dissolved in neurobasal medium at the required final concentrations in advance). After 1 min of drug treatment, the influence of the drug on the frequency and amplitude of the neuronal calcium oscillations was observed for 2 min. The images during the complete process were collected using the laser scanning confocal microscope. All of the results were analyzed using the TCS-SP2 Confocal Laser Scanning Microscope (CLSM) software. The pretreatment and intervening drugs included ketamine, MK801, and NMDA.

### Quantitative Analysis of the Frequency and Amplitude of the Calcium Oscillations in Hippocampal Neurons

A semi-quantitative method was used for the quantitative analysis of the calcium peaks in the neuronal calcium oscillation. This method used the changes in the intracellular calcium fluorescence (Fluo-4AM, F/F_0_) to represent the changes in the cytosolic calcium concentrations, where F is the cytosolic calcium fluorescence value at time t and F_0_ is the arithmetic mean of the minimum cytosolic fluorescence values ±10 s of time t; F/F_0_>1.2 was defined as the occurrence of one calcium oscillation. Based on the characteristics of the neurons, 5 neurons in each field were selected for observation. All of the experiments (starting from cell culture) were repeated three times.

### Data Analysis

All of the data are presented as 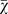
±*s* and were analyzed using the SPSS16.0 software. The frequency and amplitude of the calcium oscillations were statistically analyzed using the paired Wilcoxon rank sum test and the paired t-test, respectively. Repeated measures were used for the reversal effect of NMDA on the ketamine-induced inhibition of the calcium oscillations. The Friedman method was used for the statistical analysis of the frequency. *p*<0.05 indicated that the difference was statistically significant.

## Results

**Figure 1:**
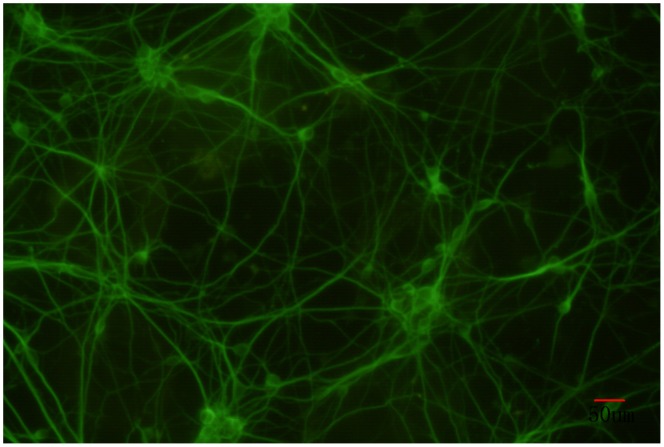
Hippocampal neurons cultured on the fifth day *in vitro* with immunofluorescence staining of β-tublinШ.

**Figure 2:**
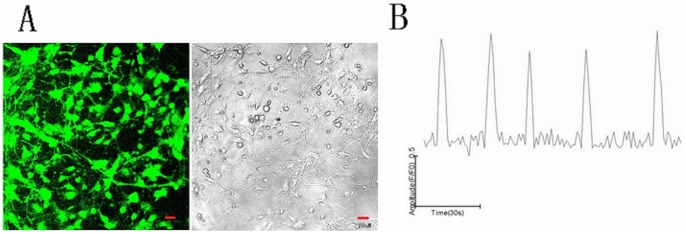
Ca^2+^ fluorescent and Ca^2+^ oscillation images of hippocampal neural cells. A: Imaging under a laser scanning confocal microscope using Fluo-4 AM calcium fluorescence probes. B: Representative images of the calcium oscillations in *in vitro* cultured hippocampal neurons on day 5 (the scales for the calcium oscillation amplitude and time are shown).

After 5 days of *in vitro* culture, the axons and dendrites of the hippocampal neurons had developed into a sparse network that primarily formed an *in vitro* neural network and effective synaptic connections (Fig. 1). After these were stained with the Fluo-4AM calcium fluorescence dyes and imaged under a laser scanning confocal microscope, the hippocampal neurons were randomly selected for observation. The results showed that the typical calcium oscillations were present. In these neurons, the calcium oscillation frequency was 0.042±0.006 Hz and the amplitude (F/F_0_) was 2.01±0.07 (Fig. 2).

**Figure 3:**
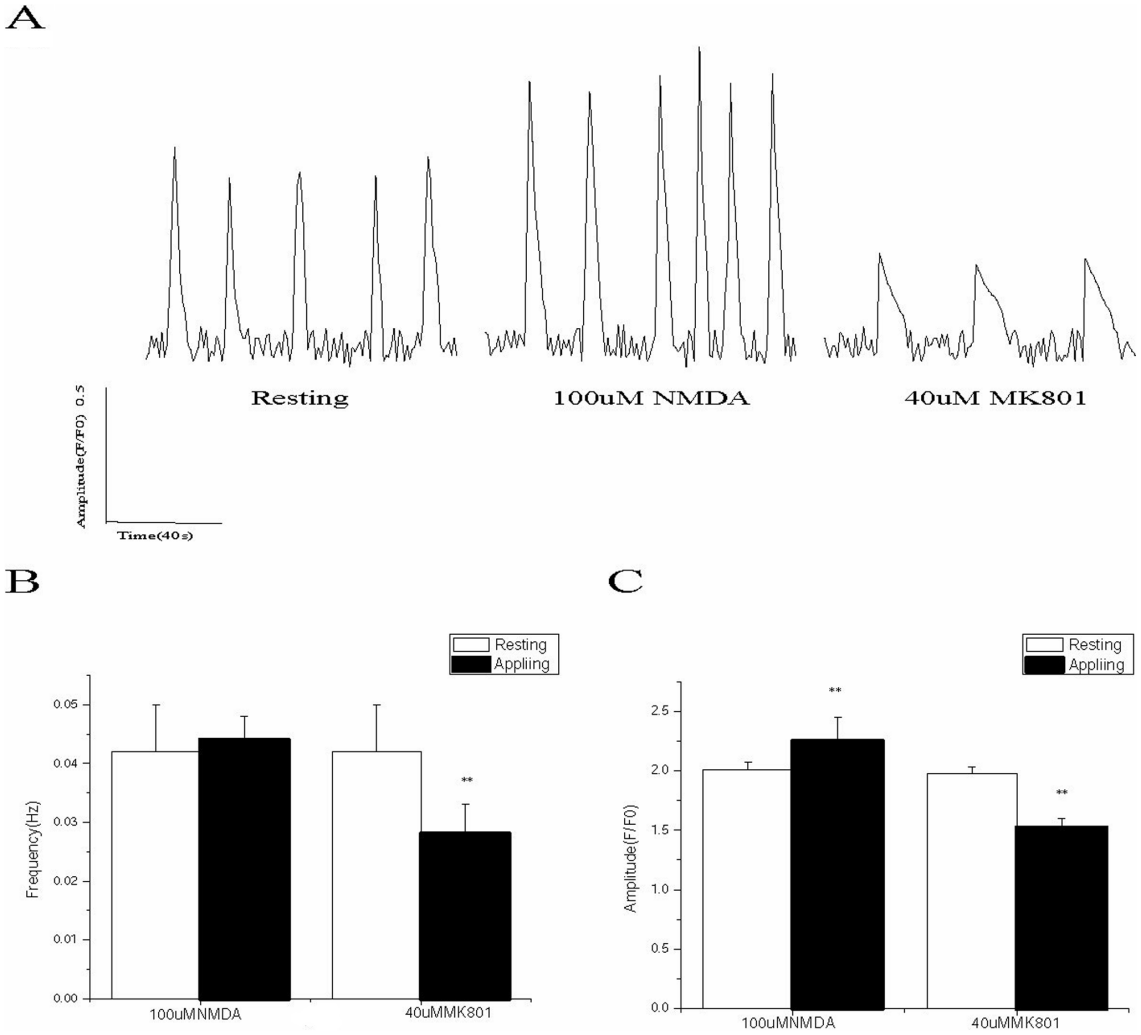
Effects of 100 µM NMDA and 40 µM MK801 on the frequency and amplitude of the calcium oscillations in developing hippocampal neurons. A: Representative images of the calcium oscillations during the pre-intervention phase, the post-treatment phase of 100 µM NMDA, and the post-treatment phase of 40 µM MK801. The application of NMDA significantly increases the amplitude of the calcium oscillations (**p<0.01); however, its effect on the frequency was not statistically significant. MK801 significantly inhibited the amplitude and frequency of the calcium oscillations (**p<0.01). B and C: Effects of NMDA and MK801 on the calcium oscillations.

Using a final concentration of 100 µM NMDA for the intervention strengthened the amplitude of the neuronal calcium oscillations. In addition, the change in the F/F_0_ values was statistically significant. However, although the oscillation frequency increased slightly, the difference was not statistically significant compared with the frequency obtained in other groups prior to the intervention. The non-competitive NMDAR antagonist, MK-801, at a final concentration of 40 µM significantly reduced the amplitude and frequency of the calcium oscillations in the hippocampal neurons; these differences were statistically significant (Fig. 3).

**Figure 4:**
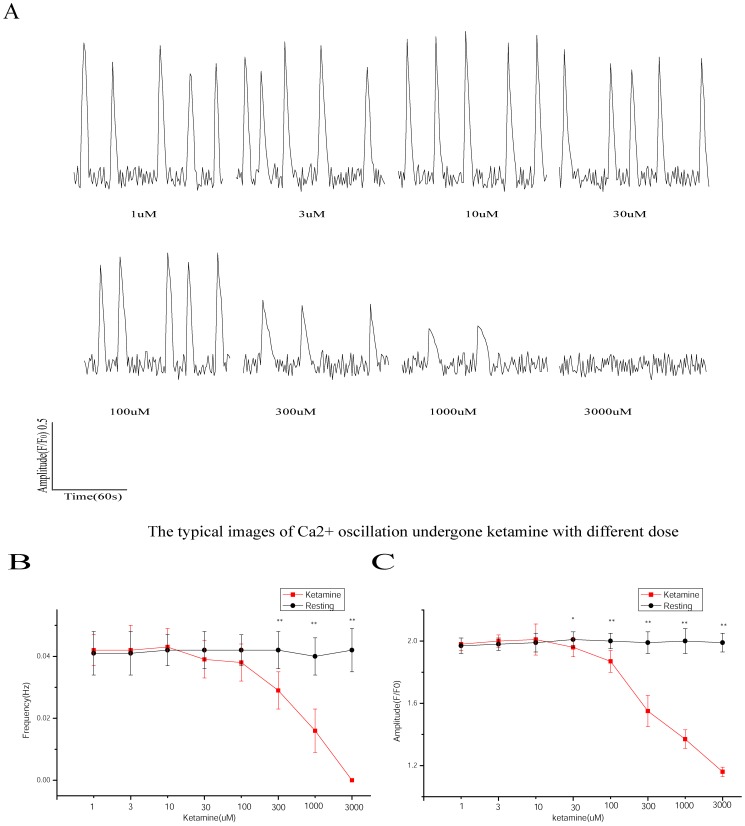
Effects of different concentrations of ketamine on the frequency and amplitude of the calcium oscillations in developing hippocampal neurons. A: The typical images of Ca^2+^ oscillation were undergone with different concentrations.An increase in the ketamine concentration from 30 µM inhibited the calcium oscillations in a dose-dependent manner. B: Ketamine concentrations greater than or equal to 300 µM decreased the frequency of the calcium oscillations compared with the frequency measured before the drug treatment (**p<0.01). C: Ketamine concentrations greater than or equal to 30 µM decreased the amplitude of the calcium oscillations compared with the amplitude measured before the drug treatment (**p<0.01).

A concentration of ketamine in the range of 1–10 µM did not significantly affect the frequency and amplitude of the calcium oscillations in developing hippocampal neurons. Increases in the ketamine concentration, starting from 30 µM, gradually decreased the amplitude of the neuronal calcium oscillations. However, although 30–100 µM ketamine could inhibit the frequency of the calcium oscillations, the results were not statistically significant. Ketamine concentrations starting from 300 µM inhibited the frequency of the calcium oscillations; these results were statistically significant. A ketamine concentration of 3,000 µM completely abolished the neuronal calcium oscillations (Fig. 4).

**Figure 5:**
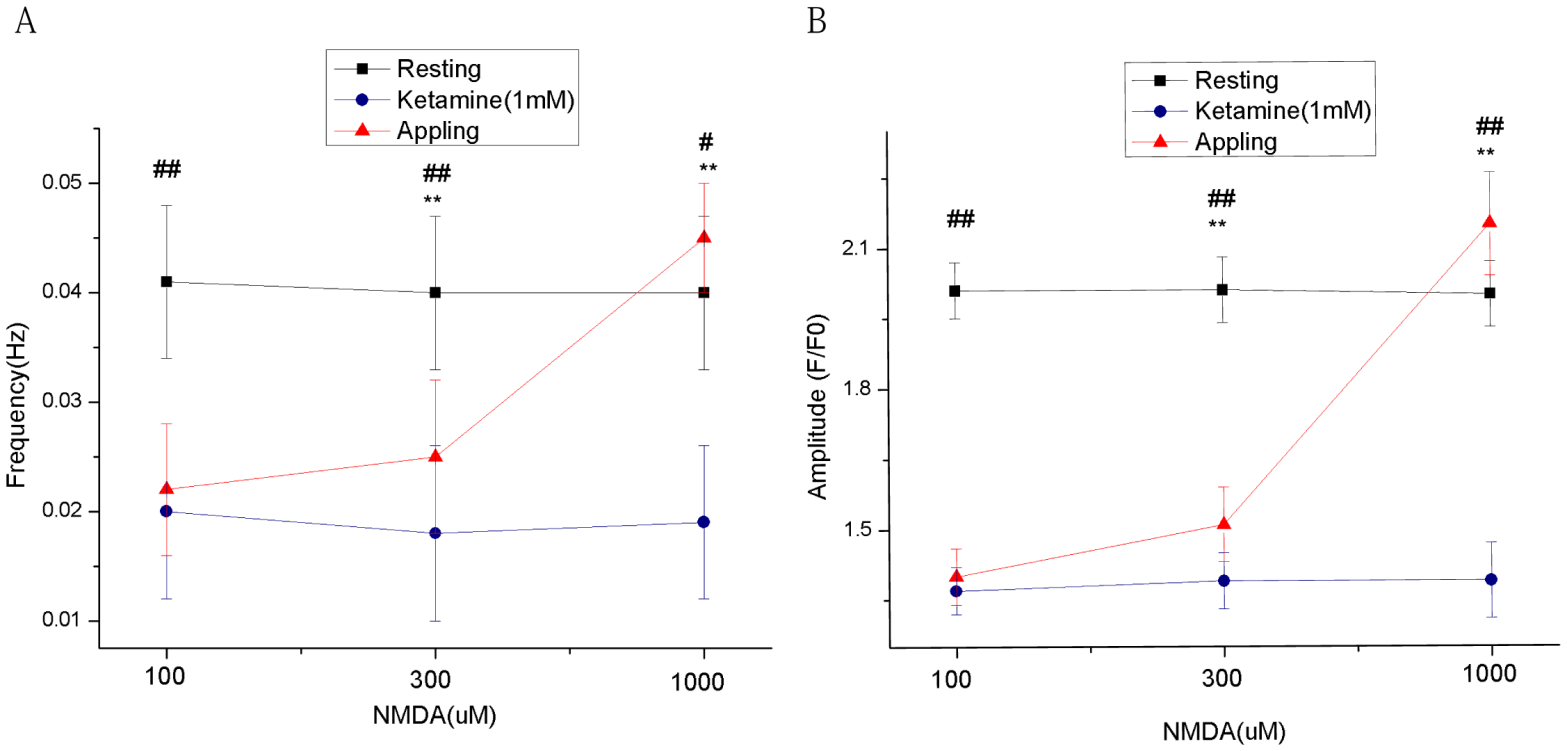
The reversal effects of different concentrations of NMDA on the ketamine-inhibited calcium oscillations. A and B: The frequency and amplitude of the calcium oscillations before drug intervention, after treatment with 1 mM ketamine, and after treatment with different concentrations of NMDA. The calcium oscillations inhibited by 1 mM ketamine were gradually recovered with an increase in the NMDA concentration. The administration of 1,000 µM NMDA not only restored the frequency and amplitude of the calcium oscillations but also resulted in a frequency and amplitude of the oscillations that exceeded those measured before the drug intervention. (^#^ *p*<0.05,^##^ *p*<0.01 vs resting condition; **p<0.01 vs 1 mM Ketaminecondition).

NMDA concentrations of 100, 300, and 1,000 µM were used to treat the hippocampal neurons that were pretreated with 1 M ketamine. The frequency and amplitude of the calcium oscillations gradually reversed with an increase in the NMDA concentration; when the NMDA reached 1,000 µM, the frequency and amplitude of the calcium oscillations were completely reversed and exceeded both the frequency and amplitude that were observed before the ketamine treatment (Fig. 5).

## Discussion

Calcium oscillations comprise a ubiquitous physiological phenomenon that plays an important role in the physiological developmental processes of the nervous system, such as the migration and differentiation of neurons and the establishment of synaptic connections. At the early stage of neuronal development, calcium oscillations already occur; this oscillation signal can regulate the early maturation and developmental processes of the nervous system [13]. Studies using immature spinal cord neurons have shown that intracellular calcium transients with different frequencies can promote the expression of neurotransmitters, the maturation of channels, and neurite growth; in addition, the inhibition of the calcium influx can impede neuronal differentiation [14]. During the migration process of *in vitro* cultured cerebellar granule cells, the frequency and amplitude of the calcium oscillations play crucial roles. The application of calcium channel blockers to block the calcium influx can inhibit both the frequency and the amplitude of the calcium oscillations and thus results in the delayed migration of the cerebellar granule cells; this delayed migration can be restored when the frequency and amplitude of the calcium oscillations are recovered [15]. In transgenic neurons and neuroendocrine cells, it has been shown that a change in the Ca^2+^ in the synaptic vesicles regulates the expression of synaptotagmin 1, which then regulates the release of neurotransmitters [16]. These results all indicate that the frequency and amplitude of the calcium oscillations and their temporal and spatial expression diversity plays pivotal roles in the developmental processes of the nervous system. Cytosolic calcium oscillations that exceed a certain temporal and spatial scope will trigger the apoptosis or necrosis of neurons, thus influencing neuronal death [17], [18].

The hippocampus exhibits a relatively dense and independent neuronal distribution and is involved in many complex physiological functions that rely on the establishment of effective synaptic connections, such as learning, memory, and emotional reaction. Based on previous study results, a period of 3–5 days is the critical period for the establishment of synaptic connections in *in vitro* cultured hippocampal neurons. In this period, neurons are at their most sensitive to external stimuli, including the neurotoxicity of anesthetics, and are most susceptible to damage [19]. Therefore, we used hippocampal neurons that were cultured *in vitro* for 3–5 days as our study subjects to observe the effect of ketamine on the characteristics of the calcium oscillations of these cells.

In primary cultured hippocampal neurons, the calcium oscillation activity is associated with dynamic changes in the membrane potential and has a very complex formation mechanism. Studies have shown that one calcium oscillation consists of a series of steps [20]–[23]: 1) the extracellular Ca^2+^ enters the cytosol through the simultaneous activation or the synergistic effect of ligand-gated (such as NMDAR) and voltage-sensitive calcium channels; 2) the Ca^2+^ that enters the cytosol can produce a calcium-releasing effect to induce the release of Ca^2+^ from the endoplasmic reticulum (ER) through the activation of inositol 1,4,5-triphosphate receptors (IP_3_R); 3) a portion of the released Ca^2+^ is uptaken by the mitochondria, and the mitochondrial Ca^2+^ can enter the cytosol through the Na^+^-Ca^2+^ exchanger to induce the release of calcium from the ER until the mitochondrial calcium is depleted; 4) after the messenger effects are completed, the increased cytosolic Ca^2+^ will be rapidly exported outside the cells by Ca^2+^-ATP pumps and Na^+^-Ca^2+^ exchangers on the cell membrane or rapidly removed by Ca^2+^-ATPase in the ER; and therefore, 5) the cytosolic Ca^2+^ level returns to normal. However, because different laboratories have obtained contradictory results, it is still controversial whether NMDAR plays a decisive role in the formation of calcium oscillations [9], [24], [25]. The frequency and amplitude of the calcium oscillations are found to be different among different types of excitatory cells, among cells that are cultured with different culture methods, among cells in different developmental stages, and due to different measurement methods. Our study showed that the frequency and amplitude (F/F_0_) of the calcium oscillations in immature hippocampal neurons was 0.042±0.006 Hz and 2.01±0.07, respectively. This study showed that 100 µM MNDA did not have any statistically significant effect on the frequency of the calcium oscillations, but did induce a significant increase in the amplitude of the calcium oscillations. In addition, the application of 40 µM MK801 could induce a significant inhibition of the amplitude and frequency of the calcium oscillations. However, Sinner et al. demonstrated that the same dose of MK801 caused a complete inhibition of the calcium oscillations [26]. We hypothesize that, in addition to the differences in the methods used for the cell culture and the measurement of the calcium oscillations, this inconsistency is mainly due to the different developmental stages of the study subjects. The cells used in our study were at the critical period for the formation of synaptic connections; these cells were neurons at the rapid developmental stage, and the experiment was performed on the fifth day of *in vitro* culture. In contrast, Sinner et al. used hippocampal neurons cultured for 17–18 days *in vitro*; at this time, the hippocampal neurons are substantially mature and/or nearly aging. NMDAR is a heterotetramer that consists of different types of subunits: NR1, NR2, and NR3. During different developmental stages of the central nervous system, the expression of NMDAR subunits is also different [27], [28]. For example, the expression of the NR1 subunit can be found in 14-day-old rat embryos, reaches its peak in postnatal week 3, and then gradually decreases over time to the adult level [29]. The expression of NR2 reaches its peak in postnatal week 3 and is then gradually reduced to the adult level [30]. NR3 is mainly expressed in the developing central nervous system, which also exhibits temporal scalability [31]. Different NMDAR subtypes are involved in different physiological processes [32], [33]; this may thus be the main reason responsible for the different experimental results. Our experimental model confirmed that NMDARs definitely participate and play a pivotal role in the formation of cytosolic calcium oscillations in neurons. These results provide an experimental basis for our subsequent studies on another intravenous anesthetic, ketamine, which is a non-competitive NMDA antagonist.

Many recent studies have demonstrated that the repeated application of ketamine or MK801 during the rapid developmental stage of the nervous system can affect the development of the nervous system, induce increased neuronal apoptosis, and even affect the learning and memory functions observed in adulthood [11], [12], [34]. In this study, we investigated the effects of different concentrations of ketamine on the frequency and amplitude of the calcium oscillations. The lowest ketamine concentration that we used was 1 µM, and we increased this dosage three-fold until we obtained the maximum concentration of 3,000 µM. We found that the effect of 1–10 µM ketamine on the frequency and amplitude of the calcium oscillations was not statistically significant. However, it is worth noting that, within this concentration range, the frequency and amplitude of the calcium oscillations exceeded those measured before the drug application. This result was similar to that reported by Sinner et al.: the administration of 3 µM of the R (−) isomer of ketamine noticeably increased the calcium oscillation frequency, but this difference was not statistically significant [26]. When the ketamine concentration reached 30 µM, the amplitude of the calcium oscillations was gradually inhibited in a dose-dependent manner. However, the inhibition of the calcium oscillation frequency occurred and showed statistical significance only when the ketamine concentration reached 300 µM. The inhibition of the calcium oscillations by ketamine was dose dependent. When the ketamine concentration increased to 3,000 µM, the calcium oscillations were completely eliminated. Studies have shown that the blood concentration of ketamine that has an anesthetic effect on rats is approximately 20 µM [35]. Because ketamine can easily pass through the blood-brain barrier due to its high lipid solubility and because the cerebral blood flow can also be increased by ketamine, the ketamine concentration in the cerebrospinal fluid (CSF) is 6.5-fold that found in blood [36]. Thus, a ketamine concentration of 100–200 µM is similar to the clinical level. The administration of ketamine within this concentration range significantly inhibited the amplitude of the calcium oscillations in the developing hippocampal neurons; however, the inhibition of the frequency was not statistically significant, and the calcium oscillations were not completely inhibited. The administration of ketamine within the clinical concentration range can downregulate the opening time of the channels, decrease the opening frequency of the channels, and inhibit the Ca^2+^ influx through the NMDAR channels through an allosteric mechanism when the NMDAR channels are open [37], [38]. The administration of ketamine at concentrations that are higher than the clinical concentration of 300 µM can significantly inhibit the frequency and amplitude of the calcium oscillations in a dose-dependent manner. This substantial inhibition may be associated with the fact that the opening time and frequency of the NMDAR channels gradually decreases with an increase in the ketamine concentrations as well as the fact that a high dose of ketamine also inhibits the GABA_A_ receptor [39]. Different from its function in mature neurons, GABA is an excitatory neurotransmitter during the early developmental process, especially within a few days to one week after birth. The GABA_A_ receptor can mediate the membrane potential depolarization, activate the voltage-sensitive Na^+^ and Ca^2+^ channels, and increase the NMDAR activities through the suppression of the inhibitory effect of Mg^2+^. Therefore, the extracellular Ca^2+^ that enters the cells through the NMDAR channels or the voltage-sensitive L-type calcium channels leads to increases in both the amplitude and the frequency of the calcium oscillations [5], [40]. In contrast, if the concentration of ketamine used exceeds the clinical dose, the GABA_A_ receptor will be inhibited and the Ca^2+^ influx will decrease, thereby inhibiting the calcium oscillations. All of the above hypotheses might be responsible for the ketamine-induced inhibition of the calcium oscillations in developing hippocampal neurons. In our study, a ketamine concentration of 3,000 µM completely inhibited the neuronal calcium oscillations, which is inconsistent with the finding of Sinner et al., who reported that 250 µM ketamine can completely inhibit the occurrence of calcium oscillations [26]. These results indicate that developing hippocampal neurons are more resistant to the inhibition of their calcium oscillations by ketamine than mature hippocampal neurons.

In subsequent experiments, we administered different concentrations of NMDA to neurons that had been inhibited by 1 M ketamine to investigate whether NMDA could reverse the ketamine-induced inhibition of the neuronal calcium oscillations. We showed that 100 µM NMDA was not sufficient to reverse the ketamine-induced inhibition of the neuronal calcium oscillations; however, an increase in the NMDA concentration gradually exhibited a reversal effect. An NMDA concentration of 300 µM showed a partial effect. However, when the NMDA concentration reached 1 mM, in addition to the inhibitory effect of ketamine on the neuronal calcium oscillations being completely reversed, the frequency and the amplitude of the calcium oscillations exceeded the levels observed before the administration of ketamine. These results may be associated with the increased number and frequency of the opening NMDAR channels. When the NMDAR channels are open, the Ca^2+^ influx increases and the cell membrane depolarizes; these effects will further activate the voltage-dependent calcium channels, increase the amount and frequency of the Ca^2+^ influx, and induce the releasing effect of the intracellular calcium storage, thereby resulting in an increase in the frequency and amplitude of the calcium oscillations.

Ketamine inhibits the calcium oscillations in developing hippocampal neurons in a dose-dependent manner; in addition, a clinical concentration of ketamine inhibits the amplitude of the calcium oscillations. Because the alterations in the frequencies and amplitudes of the calcium oscillations encode important information, the ketamine-induced inhibition of the calcium oscillations in developing hippocampal neurons will substantially affect the growth and development of these neurons and even cause apoptosis.
